# Who is afraid of Christmas? The effect of Christmas and Easter holidays on psychiatric hospitalizations and emergencies—Systematic review and single center experience from 2012 to 2021

**DOI:** 10.3389/fpsyt.2022.1049935

**Published:** 2023-01-11

**Authors:** Else Schneider, Timur Liwinski, Lukas Imfeld, Undine E. Lang, Annette B. Brühl

**Affiliations:** University Psychiatric Clinics Basel, Clinic for Adults, University of Basel, Basel, Switzerland

**Keywords:** emergency care, hospitalization, Christmas, Easter, emergency psychiatric care, admission

## Abstract

**Background:**

Major holidays such as Christmas and New Year’s Eve are regular occasions for get-togethers in families and other social groups. Socially, these days are often loaded with memories and expectations but also involve the potential for interpersonal tension and conflicts and disappointments. In addition, loneliness might also be most intense during these days. All these factors might lead to the expectation of increased mental distress and subsequently increased help-seeking in psychiatric contexts resulting in emergency psychiatric contacts, psychiatric hospitalizations, and even suicidal behavior. But is there evidence for increased psychiatric emergencies and hospitalizations around the days of Christmas?

**Methods:**

The existing evidence is systematically reviewed here (studies in PubMed in English investigating annual and Christmas-related variations in suicide (attempts), psychiatric emergencies and hospitalizations, last search date (13.07.2022) and complemented by an analysis of acute admissions at the University Psychiatry Clinics Basel, Switzerland, around Christmas and Easter holidays compared to the other days of the year. Easter was chosen as a comparison holiday.

**Results:**

In 25 reviewed studies, Christmas holidays were not associated with increased utilization of emergency psychiatric services. In contrast, hospitalizations were lower on Christmas and other holidays than the rest of the year. Analyzing the annual variation of 26,088 hospitalizations in our center between 2012 and 2021 revealed the same pattern.

**Conclusion:**

The assumption of increased utilization of psychiatric emergency services on Christmas and other major holidays is not confirmed by multiple studies around the globe in various socio-cultural and medical settings. The study is registered in the international prospective register for systematic reviews (PROSPERO; 351057).

**Systematic review registration:**

https://www.crd.york.ac.uk/prospero/, identifier 351057.

## Background

A 2019 YouGov survey reported that 36% of the British population reported that December and particularly the Christmas period has a favorable impact on their mental health and 31% reported no difference in mental health (1). Nevertheless, popular media mostly (up to 70%) focus on the 26% of the population which report a fairly or very adverse effect (2). Clinicians in leading psychiatric institutions repeatedly receive requests for comments on the assumed “Christmas surge” of emergencies, suicides, and hospitalization demands.

This assumption is based on classical psychoanalytical literature such as “Negative Reactions to Christmas” by J. Eisenbud (3) as well as S. Ferenczi’s (4) “Sunday Neuroses” and J.P. Cattell’s “The holiday syndrome” (1955) (5). But even nowadays, a professional organization such as mind.org.uk explains “Why Christmas is a hard time” and gives “Christmas coping tips”. However, the top five reasons people seek emergency departments during the holiday season, according to the Lovelace Health System, are (1) falling decorations, (2) food poisonings, (3) cuts/lacerations, (4) broken toes, and (5) problems associated with overindulgence, particularly gastrointestinal and cardiac illnesses. In cardiology and emergency services, the Christmas and New Year’s holidays is the period with the highest mortality rate because of cardiac and non-cardiac diseases compared to the rest of the year (6).

What is the evidence about mental health problems and psychiatric hospitalizations around the holiday season, particularly Christmas, New Year’s Eve, and New Year’s Day? Are there more suicides, more psychiatric emergency treatments, and more hospitalizations during the holidays?

This systematic review summarizes the evidence on this question qualitatively. It additionally analyses the daily hospitalization rates at the University Psychiatric Clinics of Basel, Switzerland, which is a major psychiatric healthcare provider offering in- and outpatient treatment primarily for the city and the canton of Basel-Stadt as well as for the neighboring cantons comprising approximately 200,500 individuals with 277 beds in the clinic for adult psychiatry and the private clinic.

## Methods

### Systematic review

We conducted the systematic review following the guidelines recommended by the Preferred Reporting Items for Systematic Reviews and Meta-Analyses (PRISMA) statement and the recommendations of the Center for Reviews and Dissemination of the University of York (7, 8).

**FIGURE 1:**
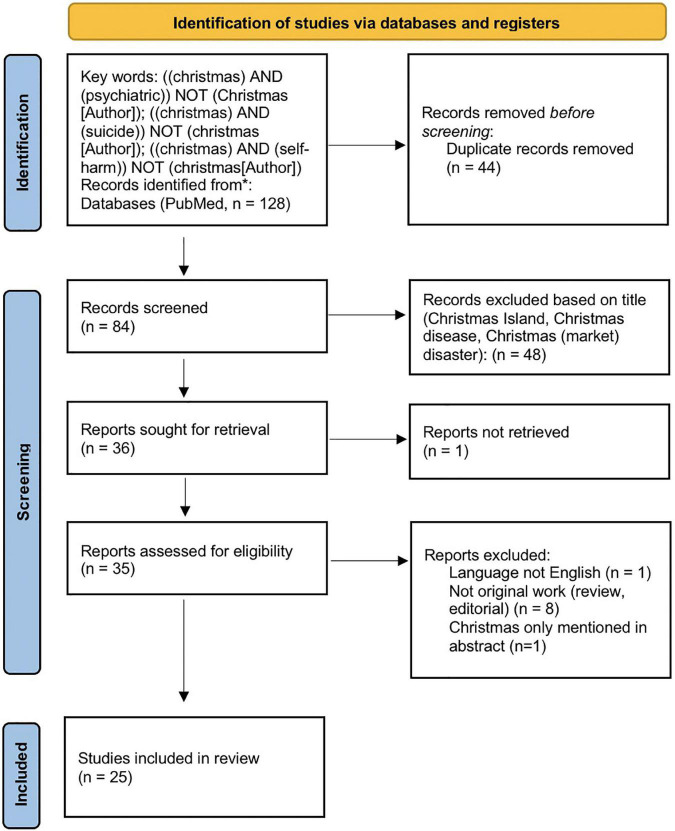
Flowchart of literature selection. Studies were first identified on 10th January 2022. Data were extracted on 11th and 12th January 2022. On 13th July 2022, another search with the same search terms was conducted to check for recent additions. Source: https://www.prisma-statement.org/PRISMAStatement/FlowDiagram.

A systematic literature survey was conducted on the 1st of October 2021 using MEDLINE (*via* PubMed) from inception to 10th January 2022, then updated on 13th July 2022, using the search terms: [(Christmas) AND (psychiatric)] NOT (Christmas [Author]); [(Christmas) AND (suicide)] NOT (Christmas [Author]); [(Christmas) AND (self-harm)] NOT (Christmas [Author]). The selection process details are shown in the PRISMA flow chart (Figure 1).

The authors surveyed the abstracts independently for eligibility. The inclusion decision was based on discussion and consensus. A manual cross-reference search was performed additionally to identify other relevant tags. Only full-length articles written in English were included. The quality of the studies, including the risk of bias, was evaluated based on the Critical Appraisal for Qualitative Studies checklist provided by the Center for Evidence-Based Medicine (CEBM) at the University of Oxford.^1^ Because of the variation in reported measures, results are presented qualitatively in Table 1. The PRISMA 2020 checklist, quality assessment and other relevant aspects to the systematic review process are included in the Supplementary material file.

**TABLE 1:** Summary of the relevant literature identified.

	References	Measure (year, period)	Peak (↑)	Trough (↓)	Christmas (↑/↓)	New year’s eve (↑/↓)
	**Studies investigating the whole year**					
1	Hofstra et al. (13)	Suicide (year)	Spring	Christmas	↓	=January
2	Su et al. (14)	Poisoning (year)	Spring	Winter	↓	=(NA)
3	Cavanagh et al. (15)	Suicide general population, clinical population	1.1. (general) 21.5. (clinical) January (general) May (clinical)	25.12. (general), Christmas (clinical) December (Oct, Nov) ↓ all	↓ (lowest in all populations)	↑ (general)
4	Fernández-Niño et al. (11)	Suicide (year)	May 1.1., 25.12.	February	25.12. ↑, other ns	1.1.: ↑
5	Plöderl et al. (16)	Suicide (year)	Spring	Christmas	↓	1.1.: ↑
6	Beauchamp et al. (17)	Suicide attempts/Poison	Spring, fall	Winter, summer	↓	1.1.: ↑
7	Ajdacic-Gross et al. (18)	Suicide (year)	May/June	December	23.–27.12.: ↓	30.12.–02.01.: ↓
8	Bollen (19)	Suicides, motor vehicle fatalities (year)	April, May	December, November, January	↓	1.1. : ↑
9	Nakamura et al. (10)	Suicide attempts adolescents (year)	January	December	↓ (NS)	NS (increase after)
10	Masterton (12)	Parasuicide (deliberate self-harm), gender (year)	May–September (women only)	December (women only)	↓ (w: lowest numbers of the year in week including 24^th^)	↓ up to 31^st^, ↑ week after
11	Velamoor et al. (20)	Psychiatric emergencies	March (NS)	December (NS)	NA (only monthly)	NA (only monthly)
12	Halpern et al. (21)	Psychiatric emergencies	NA	December (substance abuse higher, all other diagnoses lower)	↓	31.12. ↓ Increase week after
	**Studies investigating a limited time frame**					
13	Hadlaczky and Hökby (22)	Suicide (December 15–January 15)	1.1.	Christmas	↓/ =	=1.1. ↑
14	Barker et al. (23)	Suicide (holidays)	NA	NA	24.12. ↑, 25./26.12. ↓	1.1.: ↑
15	Ajdacic-Gross et al. (24)	Suicide (October-February)	4.1.	Christmas (eve)	↓	NS
16	Zonda et al. (25)	Suicide (weeks around Christmas)	NA	NA	24.12. ↓ All ↓	1.1.: ↑
17	Jessen et al. (26)	Suicide attempts around holidays	NA	NA	23.–26.12.: ↓ 27.12.: ↑	31.12.: ↓ 01.01.: ↑ Similar effect on easter, no similar effects on other public holidays
18	Phillips and Wills (27)	Suicides around major holidays	NA	NA	↓, up to 5 days pre and post	31.12.: ↓ 01.01.: ↑ (only New Year and July 4^th^ with increase after holiday)
19	Sparhawk (28)	Suicide on holidays	NA	NA	↓, including week centered around Christmas	NA
20	Griffin et al. (29)	Self-harm (holidays)	NA	NA	Women: NS/↑, Men: ↑/NS	1.1. ↑
21	Bergen and Hawton (30)	Deliberate self-harm (December 16–January 6)	NA	NA	24.–26.12.: ↓ (women > men)	31.12. ↓/NS 1.1.: ↑/NS
22	Cullum et al. (31)	Deliberate self-harm (Christmas, Valentine’s day, control days)	NA	NA	↓ (negative association)	NA
23	Hillard (32)	Emergencies (weeks around Christmas)	2–4 weeks after Christmas	Week before Christmas	↓	↓
24	Sauer et al. (33)	Psychiatric emergencies (around Christmas and New year)	NA	NA	↓ (increase after holidays)	↓ (increase after holidays)
25	Ballard et al. (34)	Hospitalization (Christmas census)	NA	NA	Lower than in February	NA

↑ increased, ↓ decreased, NA, not available; NS, not significant.

### Original data from the University Psychiatric Clinics Basel

We performed a retrospective longitudinal cohort study on the internal register of the University Psychiatric Clinics (UPK) Basel, Switzerland. The UPK are a part of the public health system in Basel and are among the leading psychiatric clinics in Switzerland. There are approximately 300 beds available in four specialist clinics. The UPK provide the full range of psychiatric and psychotherapeutic care. Access to mental health services in the catchment area is available with a low hurdle as medical insurance is obligatory for all the country’s inhabitants and covers nearly all treatment costs.

Data processing and statistical analyses were performed in R (v4.4.2; R Foundation for Statistical Computing, Vienna, Austria). The Statement of the Strengthening the Reporting of Observational Studies in Epidemiology (STROBE) was used as a standard for the complete and transparent reporting of observational data. Poisson regression analysis was used to explore time trends in psychiatric hospital admissions. In addition, we employed a Bayesian change point analysis using the Barry and Hartigan product partition model for the normal errors change point problem using Markov Chain Monte Carlo. The Bayesian analysis was implemented using the “bcp” package (v4.0.3) (9). The independent variables were day, month, year, season, Christmas days (December 25 [Christmas Day] and December 26 [St. Stephen’s Day]), Easter holidays (Good Friday, Easter Sunday, Easter Monday), and non-Christmas/Easter days. These holidays are typically non-working days often spent with family and friends. The two blocks (Christmas and Easter) have the same duration and a similar, originally Christian cultural but also societal “wrapping” or charging (specific preparations, societal presence also in shops and decorations, expectations, and anticipations). Therefore, they were chosen for comparison. The dependent variables were the daily hospital admissions numbers (total and emergency admissions only). The level of statistical significance was set at 0.05. Since we tested only a few hypotheses grounded in the reviewed empirical literature, no *p*-value correction procedure was required.

## Results

### Systematic literature review

Among the 25 included studies (Table 1), 12 addressed patterns throughout the year, including 1,499,608 subjects/cases. Six of these were done in the USA, one in Central America, and five in Europe. Ten measured suicide/suicide attempts, self-harm, and two psychiatric emergencies. The other 13 studies investigated specific patterns around the time of Christmas or holidays in general. They involved at least 339,139 subjects or incidents, with four studies not giving absolute numbers. Eight were conducted in Europe, three in the USA, one in Australia, and one involved observations in Great Britain, Australia, and Nigeria. Of those, eight addressed suicides/suicide attempts, four deliberate self-harm/parasuicide, and three psychiatric emergencies/hospitalizations. For details of the included studies, please refer to the Supplementary material.

Across the whole year cycle, all studies consistently found lower incidences of suicides/suicide attempts and psychiatric emergencies in winter. The annual peak was less consistent; most studies found it in spring, some in May/June. In the only study on adolescents, the peak was in January after the annual trough in December (10). In these general studies, one out of nine (performed in Mexico) found a higher incidence of suicides on 25th December (11). In contrast, all other studies found low and, in some, even the lowest annual incidences of suicides, suicide attempts, self-harm, or psychiatric emergencies during Christmas (i.e., 24th to 26th December). One study from Edinburgh found these effects only for women (12). For New Year, the pattern was more mixed: four studies found an increase on New Year’s Day, 1st January, three found no significant effect, and two described a reduction. One reported only monthly aggregated effects and could not differentiate between the two holiday periods.

When investigating only holidays or the specific effects around Christmas, all eight studies on suicide or suicide attempt found reduced incidences for the Christmas holidays, with some mild variations (one study found a higher incidence on the 24th followed by lower incidences on the 25th and 26th). Regarding self-harm, the effects were mixed. One study found a partially significant increase with different effects by gender, whereas the other two reported a reduction on Christmas. In all three studies, psychiatric emergencies and hospitalizations were clearly lower on Christmas. New Year was not investigated in one study. In the other 12 studies, the consistent finding was an increase in incidence on 1st January 31st December was in some studies associated with lower incidences, in some with no significant effect.

### Results from the university psychiatric clinics Basel

#### Psychiatric hospital admission trends in Basel-Stadt over the period 2012 to 2021

**FIGURE 2:**
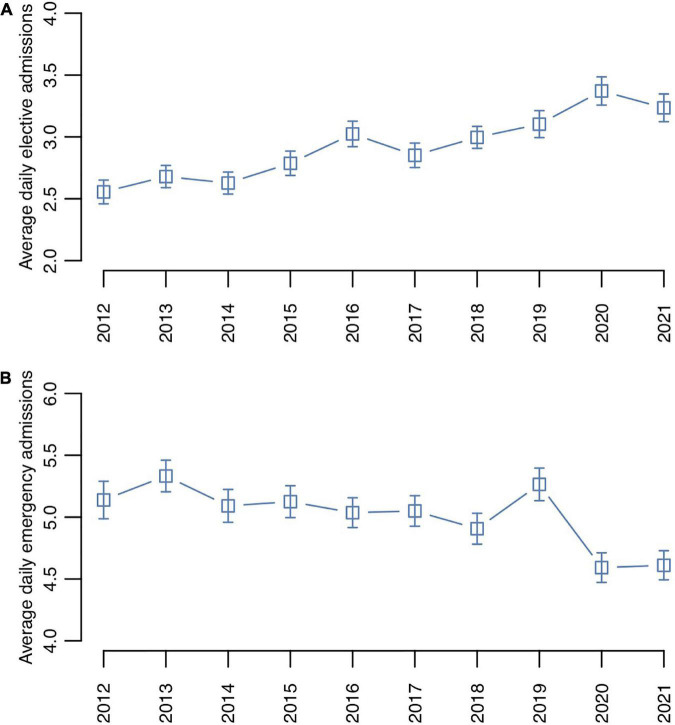
Average daily psychiatric hospitalization numbers per year: **(A)** elective admissions, **(B)** emergency admissions. The bars represent standard errors.

From 2012 to 2021, 26,088 psychiatric hospitalizations, including 18,044 emergency hospitalizations, were recorded in our center. The mean (M) daily number of admissions for the entire study period was 7.20 (standard deviation [SD] = 3.43); the average number of emergency admissions was 5.0 (SD = 2.44). We observed a slight but steady increase in average daily elective hospital admissions over the years, with 2012 being the year with the lowest average elective entries (*M* = 2.56, SD = 1.61) and 2021 the year with the highest average elective admissions (*M* = 3.37, SD = 1.90), representing an increase of 24.0% (Figure 2A).

A Poisson regression analysis of the 10-year study period indicated a statistically significant variation in the daily counts of emergency admissions between the years (χ^2^ = 20.47, *p* < 0.001; Figure 2B). Bayesian change-point analysis on the daily distribution identified a peak in the posterior probability (PP) of change points in the year 2019 (PP = 78.2%), after which the average number of daily emergency hospitalizations declined, potentially because of the ensuing COVID-19 pandemic (35).

#### Seasonal trends

**FIGURE 3:**
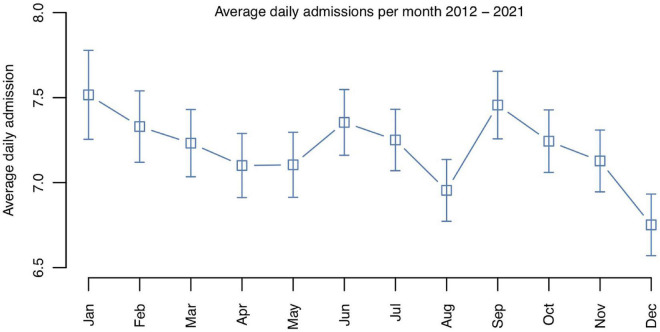
Average daily psychiatric hospitalization numbers per month over the entire study period from 2012 to 2021.

Poisson regression analysis showed a statistically significant variation of the total daily hospitalization numbers by month over the entire study period from 2012 to 2021 (χ^2^ = 21.13, *p* = 0.032). The lowest average daily hospitalization number was observed in December (*M* = 6.75, SD = 3.14), whereas January showed the highest average number of daily hospitalizations (*M* = 7.52, SD = 4.60), which represented a 10.2% difference (Figure 3). Moreover, a relatively low admission rate was observed in August, followed by a relative peak in September, most explained by the summer break in Switzerland in August. Poisson regression did not show a statistically significant variation in daily psychiatric admission numbers by season (spring, summer, fall, winter; χ^2^ = 1.11, *p* = 0.775).

#### Christmas and Easter trends

**FIGURE 4:**
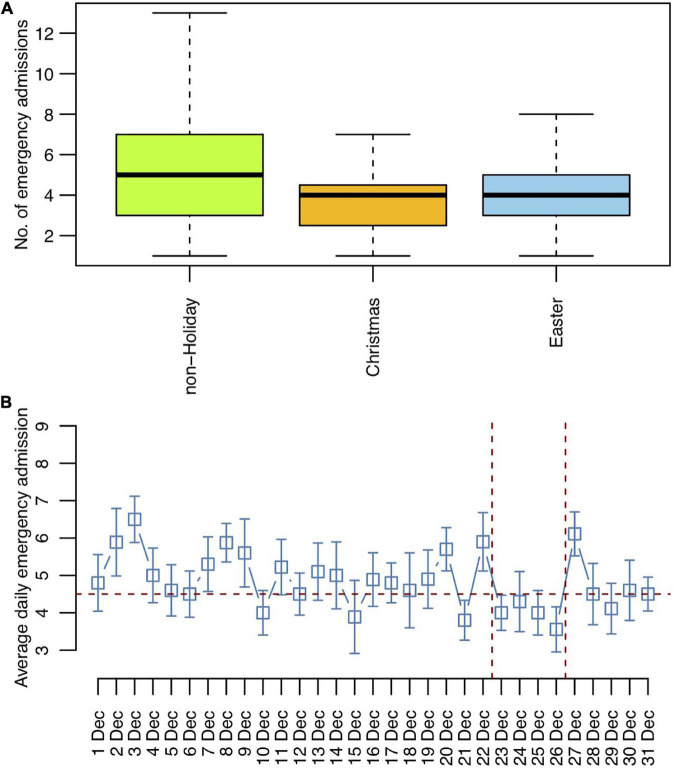
Christmas and Easter trends in emergency hospitalization trends 2012 to 2021. **(A)** Average emergency admissions per day. **(B)** Average emergency admissions per day for December with the Christmas holiday period marked.

An effect of Christmas and Easter on the daily emergency psychiatric hospital admissions was observed using Poisson regression analysis on the total study period from 2012 to 2021 (χ^2^ = 21.13, *p* < 0.001). Both Christmas (incidence rate ratio [IRR] = 0.75, standard error [SE] = 0.09, *p* = 0.016) and Easter (IRR = 0.79, SE = 0.07, *p* = 0.005) were associated with a decrease in daily emergency admission numbers compared to the other days of the year (Figure 4A). There was no statistically significant difference between the Easter and Christmas holidays (IRR = 1.05, SE = 0.15, *p* = 0.745). The average daily psychiatric emergency hospitalization number was 24.7% lower on Christmas (*M* = 3.79, SD = 1.81) and 21.1% lower on Easter (*M* = 3.97, SD = 1.81) compared to the other days (*M* = 5.03, SD = 2.45). Since December days showed overall low hospitalization numbers, we compared Christmas with the non-Christmas December days over the entire study period from 2012 to 2021. Poisson regression analysis revealed that the psychiatric hospital admission numbers were lower on the Christmas days compared to the rest of December (χ^2^ = 4.95, IRR = 0.77, SE = 0.09, *p* = 0.032), with the days from December 23rd to December 26th representing a window of constantly low average admission numbers (Figure 4B).

## Discussion and conclusion

The systematic review of the existing literature on the effects of Christmas and other seasonal factors on suicide and other psychiatric emergencies such as suicide attempts, self-harm, and emergency hospitalizations similarly showed the lowest annual measures of suicide and psychiatric emergencies in December and around Christmas, with high rates in January and, at a smaller scale, in early Summer (end of May, beginning of June).

Using our own data, we examined the daily, monthly, and seasonal trends in the numbers of psychiatric emergency patients for ten consecutive years in a major urban hospital in North-western Switzerland. The value of the present systematic review and original data lies in the empirical evidence that psychiatric emergency rates do not intensify at Christmas. On the contrary, they are significantly lower during Christmas and the Christmas season (December), with a magnitude comparable to the decrease in utilization of psychiatric emergency services during Easter. The old-fashioned lore that Christmas holidays aid and abet psychiatric exigency is, thus, not supported by the empirical data. The limitation of our original data lies in the reliance on the experience from one urban clinical center only; however, our data are broadly consistent with those gathered in the systematic literature survey. Previously, Sansone and Sansone concluded in their literature review that psychopathology displays two broader patterns during the Christmas holidays. On the one hand, there appears to be an increase in mental issues such as worsening mood and alcohol-related disorders. In contrast, the authors reported a decrease in the overall utilization of psychiatric emergency services (36). In line with previous literature, we also found a “rebound effect” with January being the month with the highest recourse to psychiatric emergency services utilization. This observation suggests that there might be a rise in dysphoric moods in the period after the protective holiday effect wanes. Future research should direct efforts at exploring coping strategies and cognitive-behavioral skills to sustain the protective holiday effect and prevent end-of-holiday derangement (37). However, Christmas and Easter holidays are not a time to be dreaded by the public and mental healthcare providers.

## Risk of bias and limitations

We found no clear evidence of biases in the included studies. All studies except one were conducted in the world’s northern hemisphere. This fact could be seen as a bias, as seasonal variations other than typically experienced in the northern temperate zone might be underrepresented. Interestingly, the one study from Mexico (11) found a similar monthly pattern as in typical four seasonal climates despite a bimodal seasonal distribution (rain vs. dry). This observation could point toward negligible weather influences. Other effects influencing the annual variation might be more important. However, studies from centers in the southern hemisphere where the seasons are flipped compared to the northern hemisphere would be necessary to look for potential other contributing factors for these annual patterns. Unfortunately, the studies from Australia only investigated limited time frames (not patterns throughout the year) and can therefore not elucidate that question.

Furthermore, the study question is focused on the Christian-influenced cultural environments. Therefore, we cannot exclude that in other cultural-religious settings, certain holidays might have a negative impact on mental health and increase suicide. One study from Türkiye, however, pointed to a similar effect of Ramadan, the holy month for Muslims involving fasting rituals and social events, with a decrease in the proportion of suicide among the forensically investigated deaths during Ramadan (38) as well as a lower number of parasuicide in Jordan during Ramadan (39) and lower hospitalization rates in a psychiatric hospital in Tunisia (40). As Ramadan involves a combination of fasting and nightly social events (and eating), two studies investigated the effects of Ramadan on the course of bipolar disorder. These two studies found opposing effects, with one showing a stable course with even improved depression scores and the other showing higher relapse rates (both manic and depressed) in fasting compared to non-fasting patients with bipolar disorder (41). Therefore, further research on cultural-religious holidays and their influences on mental health is needed.

The descriptive nature of all available studies further limits the systematic review. Controlled and/or interventional studies would be difficult to design and ethically unsustainable. Therefore, we cannot draw any causal inferences. However, as the main question was to investigate the assumption of an adverse effect of Christmas holidays on mental health, the evidence of a rather opposite effect (low numbers of suicides and other psychiatric emergencies) lends no support to this assumption or any interventions toward this direction.

## Data availability statement

The raw data supporting the conclusions of this article will be made available on request by the authors, without undue reservation.

## Author contributions

ES, TL, and AB performed literature research and selection and wrote the manuscript. TL performed the statistical analyses. LI retrieved the data from the center’s database. UL gave advice and made critical comments. All authors read and contributed to the manuscript and consented to its publication.

## Footnotes

1
https://www.cebm.ox.ac.uk/resources/ebm-tools/critical-appraisal-tools?b7d2aea6-0cac-11ed-9dd4-0a25ac88ed16
