# Supplementary material for: Who is afraid of Christmas? The effect of Christmas and Easter holidays on psychiatric hospitalizations and emergencies—Systematic review and single center experience from 2012 to 2021
Source: Front Psychiatry. 2023 Jan 11;13:1049935. doi: 10.3389/fpsyt.2022.1049935 (PMC9874097; doi:10.3389/fpsyt.2022.1049935)
Supplement: Supplementary file 1 [file Data_Sheet_1.docx]

**Supplement to “Who is afraid of Christmas? The Effect of Christmas and Easter holidays on psychiatric hospitalizations and emergencies – systematic review and single centre experience from 2012 – 2021”**

Else Schneider, *^,1^ Timur Liwinski, *^,1^ Lukas Imfeld,^1^ Undine E. Lang,^1^ Annette B. Brühl^1"^

Contents

[PRISMA 2020 Checklist 2](#_Toc109830692)

[PRISMA 2020 Abstract Checklist 5](#_Toc109830693)

[Search strings used for PUBMED www.ncbi.nlm.nih.gov/pubmed 6](#_Toc109830694)

[PICO table 6](#_Toc109830695)

[List of all included studies (alphabetical order) 6](#_Toc109830696)

[Quality assessment 9](#_Toc109830697)

## PRISMA 2020 Checklist

| **Section and Topic** | **Item #** | **Checklist item** | **Location where item is reported** |
| --- | --- | --- | --- |
| **TITLE** | | |  |
| Title | 1 | Identify the report as a systematic review. | P 1 |
| **ABSTRACT** | | |  |
| Abstract | 2 | See the PRISMA 2020 for Abstracts checklist. | Additional file |
| **INTRODUCTION** | | |  |
| Rationale | 3 | Describe the rationale for the review in the context of existing knowledge. | P 2/3 |
| Objectives | 4 | Provide an explicit statement of the objective(s) or question(s) the review addresses. | P 3 |
| **METHODS** | | |  |
| Eligibility criteria | 5 | Specify the inclusion and exclusion criteria for the review and how studies were grouped for the syntheses. | P 3 |
| Information sources | 6 | Specify all databases, registers, websites, organisations, reference lists, and other sources searched or consulted to identify studies. Specify the date when each source was last searched or consulted. | P 3 |
| Search strategy | 7 | Present the full search strategies for all databases, registers and websites, including any filters and limits used. | P 3 |
| Selection process | 8 | Specify the methods used to decide whether a study met the inclusion criteria of the review, including how many reviewers screened each record and each report retrieved, whether they worked independently, and if applicable, details of automation tools used in the process. | P 3 |
| Data collection process | 9 | Specify the methods used to collect data from reports, including how many reviewers collected data from each report, whether they worked independently, any processes for obtaining or confirming data from study investigators, and if applicable, details of automation tools used in the process. | P 3 |
| Data items | 10a | List and define all outcomes for which data were sought. Specify whether all results that were compatible with each outcome domain in each study were sought (e.g. for all measures, time points, analyses), and if not, the methods used to decide which results to collect. | P 3, 4 |
|  | 10b | List and define all other variables for which data were sought (e.g. participant and intervention characteristics, funding sources). Describe any assumptions made about any missing or unclear information. | n.a. |
| Study risk of bias assessment | 11 | Specify the methods used to assess risk of bias in the included studies, including details of the tool(s) used, how many reviewers assessed each study and whether they worked independently, and if applicable, details of automation tools used in the process. | P 3 |
| Effect measures | 12 | Specify for each outcome the effect measure(s) (e.g. risk ratio, mean difference) used in the synthesis or presentation of results. | Qualitative,  P 3, 4 |
| Synthesis methods | 13a | Describe the processes used to decide which studies were eligible for each synthesis (e.g. tabulating the study intervention characteristics and comparing against the planned groups for each synthesis (item #5)). |  |
|  | 13b | Describe any methods required to prepare the data for presentation or synthesis, such as handling of missing summary statistics, or data conversions. |  |
|  | 13c | Describe any methods used to tabulate or visually display results of individual studies and syntheses. | P 3, 4 |
|  | 13d | Describe any methods used to synthesize results and provide a rationale for the choice(s). If meta-analysis was performed, describe the model(s), method(s) to identify the presence and extent of statistical heterogeneity, and software package(s) used. | P 4 |
|  | 13e | Describe any methods used to explore possible causes of heterogeneity among study results (e.g. subgroup analysis, meta-regression). | n.a. |
|  | 13f | Describe any sensitivity analyses conducted to assess robustness of the synthesized results. | n.a. |
| Reporting bias assessment | 14 | Describe any methods used to assess risk of bias due to missing results in a synthesis (arising from reporting biases). | P 3, P 11 |
| Certainty assessment | 15 | Describe any methods used to assess certainty (or confidence) in the body of evidence for an outcome. | n.a. |
| **RESULTS** | | |  |
| Study selection | 16a | Describe the results of the search and selection process, from the number of records identified in the search to the number of studies included in the review, ideally using a flow diagram. | P 11  (figure 1) |
|  | 16b | Cite studies that might appear to meet the inclusion criteria, but which were excluded, and explain why they were excluded. |  |
| Study characteristics | 17 | Cite each included study and present its characteristics. | P 6-8  (table 1) |
| Risk of bias in studies | 18 | Present assessments of risk of bias for each included study. |  |
| Results of individual studies | 19 | For all outcomes, present, for each study: (a) summary statistics for each group (where appropriate) and (b) an effect estimate and its precision (e.g. confidence/credible interval), ideally using structured tables or plots. | P 6-8  (table 1) |
| Results of syntheses | 20a | For each synthesis, briefly summarise the characteristics and risk of bias among contributing studies. |  |
|  | 20b | Present results of all statistical syntheses conducted. If meta-analysis was done, present for each the summary estimate and its precision (e.g. confidence/credible interval) and measures of statistical heterogeneity. If comparing groups, describe the direction of the effect. | Table 1 |
|  | 20c | Present results of all investigations of possible causes of heterogeneity among study results. |  |
|  | 20d | Present results of all sensitivity analyses conducted to assess the robustness of the synthesized results. |  |
| Reporting biases | 21 | Present assessments of risk of bias due to missing results (arising from reporting biases) for each synthesis assessed. |  |
| Certainty of evidence | 22 | Present assessments of certainty (or confidence) in the body of evidence for each outcome assessed. | supplement |
| **DISCUSSION** | | |  |
| Discussion | 23a | Provide a general interpretation of the results in the context of other evidence. | P 10 |
|  | 23b | Discuss any limitations of the evidence included in the review. | P 10 |
|  | 23c | Discuss any limitations of the review processes used. | P 10 |
|  | 23d | Discuss implications of the results for practice, policy, and future research. | P 10 |
| **OTHER INFORMATION** | | |  |
| Registration and protocol | 24a | Provide registration information for the review, including register name and registration number, or state that the review was not registered. | P 11 |
|  | 24b | Indicate where the review protocol can be accessed, or state that a protocol was not prepared. |  |
|  | 24c | Describe and explain any amendments to information provided at registration or in the protocol. |  |
| Support | 25 | Describe sources of financial or non-financial support for the review, and the role of the funders or sponsors in the review. | P 11 |
| Competing interests | 26 | Declare any competing interests of review authors. | P 11 |
| Availability of data, code and other materials | 27 | Report which of the following are publicly available and where they can be found: template data collection forms; data extracted from included studies; data used for all analyses; analytic code; any other materials used in the review. | n.a. |

## PRISMA 2020 Abstract Checklist

| **Section and Topic** | **Item #** | **Checklist item** | **Reported (Yes/No)** |
| --- | --- | --- | --- |
| **TITLE** | | |  |
| Title | 1 | Identify the report as a systematic review. | Yes |
| **BACKGROUND** | | |  |
| Objectives | 2 | Provide an explicit statement of the main objective(s) or question(s) the review addresses. | Yes |
| **METHODS** | | |  |
| Eligibility criteria | 3 | Specify the inclusion and exclusion criteria for the review. | Yes |
| Information sources | 4 | Specify the information sources (e.g. databases, registers) used to identify studies and the date when each was last searched. | Yes |
| Risk of bias | 5 | Specify the methods used to assess risk of bias in the included studies. | No, see main text |
| Synthesis of results | 6 | Specify the methods used to present and synthesise results. | No |
| **RESULTS** | | |  |
| Included studies | 7 | Give the total number of included studies and participants and summarise relevant characteristics of studies. | Yes |
| Synthesis of results | 8 | Present results for main outcomes, preferably indicating the number of included studies and participants for each. If meta-analysis was done, report the summary estimate and confidence/credible interval. If comparing groups, indicate the direction of the effect (i.e. which group is favoured). | Yes |
| **DISCUSSION** | | |  |
| Limitations of evidence | 9 | Provide a brief summary of the limitations of the evidence included in the review (e.g. study risk of bias, inconsistency and imprecision). | No (see main body) |
| Interpretation | 10 | Provide a general interpretation of the results and important implications. | Yes |
| **OTHER** | | |  |
| Funding | 11 | Specify the primary source of funding for the review. | n.a. |
| Registration | 12 | Provide the register name and registration number. | See main body |

*From:*  Page MJ, McKenzie JE, Bossuyt PM, Boutron I, Hoffmann TC, Mulrow CD, et al. The PRISMA 2020 statement: an updated guideline for reporting systematic reviews. BMJ 2021;372:n71. doi: 10.1136/bmj.n71 For more information, visit: <http://www.prisma-statement.org/>

## Search strings used for PUBMED [www.ncbi.nlm.nih.gov/pubmed](http://www.ncbi.nlm.nih.gov/pubmed)

((christmas) AND (psychiatric)) NOT (Christmas [Author]); ((christmas) AND (suicide)) NOT (christmas [Author]); ((christmas) AND (self-harm)) NOT (christmas[Author])

## PICO table

| **PICO** | **Inclusion Criteria** | **Exclusion Criteria** |
| --- | --- | --- |
| Population | - Humans - All ages - All genders - All races or ethnicity | - Animal studies - Non-psychiatric hospitalisations - Christmas disease |
| Intervention | - No explicit intervention (observational) | - NA |
| Comparison | - Christmas vs rest of the year - Christmas vs surrounding days | - Other holidays - No comparison |
| Outcomes | - Suicide - Suicide attempt - Deliberate self-harm - Emergency hospitalization   Secondary outcomes   - Peak & trough of outcomes throughout the year | - No outcome measure reported - Only other outcome measures reported |

## List of all included studies (alphabetical order)

- Ajdacic-Gross V, Lauber C, Bopp M, Eich D, Gostynski M, Gutzwiller F, et al. Reduction in the suicide rate during Advent—a time series analysis. Psychiatry Research 2008; 157: 139–46.
- Ajdacic-Gross V, Wang J, Bopp M, Eich D, Rössler W, Gutzwiller F. Are seasonalities in suicide dependent on suicide methods? A reappraisal. Social Science & Medicine 2003; 57: 1173–81.
- Ballard C, Bannister C, Davis R, Handy S, Cullen P, Chithiramohan R. Christmas census at a district general hospital psychiatric unit. Irish Journal of Psychological Medicine 1991; 8: 46–7.
- Barker E, O’Gorman J, de Leo D. Suicide around public holidays. Australasian Psychiatry 2014; 22: 122–6.
- Beauchamp GA, Ho ML, Yin S. Variation in Suicide Occurrence by Day and during Major American Holidays. The Journal of Emergency Medicine 2014; 46: 776–81.
- Bergen H, Hawton K. Variation in deliberate self-harm around Christmas and New Year. Social Science & Medicine 2007; 65: 855–67.
- Bollen KA. Temporal variations in mortality: a comparison of U.S. suicides and motor vehicle fatalities, 1972-1976. Demography 1983; 20: 45–59.
- Cavanagh B, Ibrahim S, Roscoe A, Bickley H, While D, Windfuhr K, et al. The timing of general population and patient suicide in England, 1997–2012. Journal of Affective Disorders 2016; 197: 175–81.
- Cullum SJ, Catalan J, Berelowitz K, O’Brien S, Millington HT, Preston D. Deliberate self-harm and public holidays: is there a link? Crisis 1993; 14: 39–42.
- Fernández-Niño JA, Astudillo-García CI, Bojorquez-Chapela I, Morales-Carmona E, Montoya-Rodriguez AA, Palacio-Mejia LS. The Mexican Cycle of Suicide: A National Analysis of Seasonality, 2000-2013. PLOS ONE 2016; 11: e0146495.
- Griffin E, Dillon CB, O’Regan G, Corcoran P, Perry IJ, Arensman E. The paradox of public holidays: Hospital-treated self-harm and associated factors. Journal of Affective Disorders 2017; 218: 30–4.
- Hadlaczky G, Hökby S. Increased suicides during new year, but not during Christmas in Sweden: analysis of cause of death data 2006–2015. Nordic Journal of Psychiatry 2018; 72: 72–4.
- Halpern SD, Doraiswamy PM, Tupler LA, Holland JM, Ford SM, Ellinwood EH. Emergency department patterns in psychiatric visits during the holiday season. Annals of Emergency Medicine 1994; 24: 939–43.
- Hillard JR. Christmas and Psychopathology. Archives of General Psychiatry 1981; 38: 1377.
- Hofstra E, Elfeddali I, Bakker M, de Jong JJ, van Nieuwenhuizen C, van der Feltz-Cornelis CM. Springtime Peaks and Christmas Troughs: A National Longitudinal Population-Based Study into Suicide Incidence Time Trends in the Netherlands. Frontiers in Psychiatry 2018; 9. doi:10.3389/fpsyt.2018.00045.
- Jessen G, Jensen BF, Arensman E, Bib-Brahe U, Crepet P, Leo D de, et al. Attempted suicide and major public holidays in Europe: findings from the WHO/EURO Multicentre Study on Parasuicide. Acta Psychiatrica Scandinavica 1999; 99: 412–8.
- Masterton G. Monthly and Seasonal Variation in Parasuicide. British Journal of Psychiatry 1991; 158: 155–7.
- Phillips DP, Wills JS. A Drop in Suicides around Major National Holidays. Suicide and Life-Threatening Behavior 1987; 17: 1–12.
- Ploderl M, Fartacek C, Kunrath S, Pichler E-M, Fartacek R, Datz C, et al. Nothing like Christmas--suicides during Christmas and other holidays in Austria. The European Journal of Public Health 2015; 25: 410–3.
- Sauer J, Ayonrinde O, Lawal R, Finn M, Ojo A. Psychiatric Emergencies and the Millennium: an International Study. International Journal of Social Psychiatry 2002; 48: 122–5.
- Sparhawk TG. Traditional Holidays and Suicide. Psychological Reports 1987; 60: 245–6.
- Su MK, Chan PY, Hoffman RS. The seasonality of suicide attempts: a single poison control center perspective. Clinical Toxicology 2020; 58: 1034–41.
- Velamoor VR, Cernovsky ZZ, Voruganti LP. Psychiatric Emergency Rates during the Christmas Season in the Years 1991 to 1997. Psychological Reports 1999; 85: 403–4.
- Zonda T, Bozsonyi Ká, Veres E, Lester D, Frank M. The Impact of Holidays on Suicide in Hungary. OMEGA - Journal of Death and Dying 2009; 58: 153–62.

Quality assessment

**Study characteristics and quality evaluation (system see below)**

| Citation | Country/region of study setting | Study design & data used | Outcome/  Measure  (year, period) & time | Max | min | Christmas (+/-) | New Year’s Eve (+/-) | comm | Quality of evidence (A, B, C) |
| --- | --- | --- | --- | --- | --- | --- | --- | --- | --- |
| **Studies investigating the whole year** | | | | | | | | | |
| Hofstra 2018^12^ | Netherlands  Europe | Whole population  National statistics | Suicide (year)  N=33’224  1995-2015 | Spring | Christmas | - | = Jan |  | A |
| Su 2020^13^ | New York City  USA | Single center  Calls to Poison Center | Poisoning (year)  Total N=257’557 cases, of which  N=42’615 suspected suicide  2009-2012 | Spring | Winter | - | = (NA) |  | AB |
| Cavanagh 2016^14^ | England (UK)  Europe | Whole population  National statistics  Suicides identified by coroners | Suicide (year)  N=73’591 suicides  1997-2012 | 1.1. (general)  21.5. (clinical)  January (general)  May (clinical) | 25.12. (general), Christmas (clinical)  December (Oct, Nov) – all | - (lowest in all populations) | + (general) |  | A |
| Fernandez-Nino 2016^10^ | Mexiko  Central America | Population  National death statistics  Suicide cases | Suicide (year)  N=64’298 cases  2000-2013 | May  1.1., 25.12. | February | 25.12. +, other ns | 1.1.: + |  | A |
| Plöderl 2015^15^ | Austria  Europe | Population  National death statistics  Suicide rate | Suicide (year + Christmas)  Total number not given  (own research: 19’153 (Austria Statistik))  2000-2013 | Spring | Christmas | - | 1.1.: + | Total number of cases not given | B |
| Beauchamp 2014^16^ | USA | Whole country  National poison data system  Reported suicidal poisoning calls to poison control centers | Suicide attempts / Poison (year, holidays)  1’065’067 exposures, of which 3’790 deaths  2006-2010 | Spring, fall | Winter, summer | - | 1.1.: + |  | A |
| Ajdacic-Gross 2003^17^ | Switzerland  Europe | Whole population  Death statistics  Death certificates | Suicide (year, Christmas)  N=37’518 suicides  1969-1994 | May/June | December | 23.-27.: - | 30.12.-02.01. - |  | A |
| Bollen 1983^18^ | USA | Whole population  National death statistics | Suicides, motor vehicle fatalities (year)  N=129’774 cases  1972-1976 | April, May | December, November, January | - | 1.1. : + |  | A |
| Nakamura 1994^9^ | Hawaii, USA  Pacific | Two hospitals, adolescents (12-18) admitted for suicide attempt | Suicide attempts adolescents (year)  N=296  1987-1991 | January | December | - (NS) | NS (increase after) |  | B |
| Masterton 1991^11^ | Edinburgh, UK  Europe | Population of Edinburgh,  regional poisoning treatment center | Parasuicide (deliberate self-harm), gender (year)  N=22’169  16 years and older  1968-1987 | May-Sept (women only) | December (women only) | - (w: lowest numbers of the year in week including 24^th^) | - up to 31^st^, + week after |  | A |
| Velamoor 1999^19^ | Southwestern Ontario  USA | Major urban hospital  Psychiatric emergency patients | Psychiatric emergencies  N=3147 (estimated)  1991-1997 | March (NS) | December (NS) | NA (only monthly) | NA (only monthly) | Only monthly numbers given | B |
| Halpern 1994^20^ | Durham, North Carolina  USA | Psychiatric evaluations Duke Medical Center, Durham | Psychiatric emergencies  Catchment area Durham county n=186’000  N=8756 visits  1987-1993 | NA | December (substance abuse higher, all other diagnoses lower) | - | 31.12. –  Increase week after |  | B |
| **Studies investigating a limited time frame** | | | | | | | | | |
| Hadlaczky 2018^21^ | Sweden  Europe | Whole population  Cause of death registry | Suicide (Dec 15 - Jan 15)  N=1047  2006-2015 | 1.1. | Christmas | -/= | =, 1.1. + |  | A |
| Barker 2014^22^ | Queensland  Australia | Queensland suicide register  Suicides recorded | Suicide (holidays)  N=10’551  1990-2009 | NA | NA | 24.12. +, 25.12.-, 26.12.- | 1.1.: + | Compared with Valentine’s & ANZAC day | A |
| Ajdacic 2008^23^ | Switzerland  Europe | Whole population  Swiss mortality statistics | Suicide (Oct-Feb)  N=49’763 suicides total,  N=19'963 Oct-Feb  1969-2003 | 4.1. | Christmas (eve) | - | NS |  | A |
| Zonda 2009^24^ | Hungary  Europe | Total population, Hungarian central statistical office,  suicides | Suicide  (weeks and days around Christmas)  Total N=133’699 suicides  1970-2002 | NA | NA | 24.12. –  All - | 1.1.: + |  | A |
| Jessen 1999^25^ | Defined catchment areas in Europe (n=12) | WHO/ EURO multicenter study on parasuicide  Reports from defined catchment areas | Suicide attempts around holidays  N=24’388  15 years or older  1989-1996 | NA | NA | 23., 24., 25., 26.: -  27.12.: + | 31.12.: -  01.01.: +  Similar effect on easter, no similar effects on other public holidays |  | AB |
| Phillips 1987^26^ | USA | National Center for Health Statistics  Reported suicides | Suicides around major holidays  Total number not given  1973-1979 | NA | NA | -, up to 5 days pre and post | 31.12.: -  01.01.: +  (only New Year and July 4^th^ with increase after holiday) |  | B |
| Sparhawk 1987^27^ | Pennsylvania  USA | Whole population  All Penn-sylvania Death certificates | Suicide on holidays, weeks before/after holiday  N=13’651  1975-1984 | NA | NA | -, including week centered around Christmas | NA |  | A |
| Griffin 2017^28^ | Ireland  EU | All emergency depart-ments, National self-harm registry | Self-harm (holidays)  N=104’371  2007-2015 | NA | NA | Women: NS/+,  Men: +/NS | 1.1. + |  | A |
| Bergen 2007^29^ | Oxford, UK  EU | All patients presenting to the Emergency Department General Hospital Oxford  Oxford monitoring system for attempted suicide | Deliberate self-harm age 10 and older  (Dec 16-Jan 6)  N=31’369 episodes  N=19’346 patients  1976-2003 | NA | NA | 24., 25., 26.12.- (women >men) | 31.12. -/NS  1.1.: +/NS |  | AB |
| Cullum 1993^30^ | London, UK  EU | 3 hospitals in Central London | Deliberate self-harm (Christmas, Valentine’s day, control days)  N not available  1983-1989 | NA | BA | - (negative assoc) | NA |  | BC |
| Hillard 1981^31^ | Durham, North Carolina  US | Catchment area 150’000 people  Duke University Medical Center ER  all psychiatric emergencies | Emergencies (weeks around Christmas)  Total N not reported  1972-1979 | 2-4 weeks after Christmas | Week before Christmas | - | - |  | BC |
| Sauer 2002^32^ | London UK  EU  Perth  Australia  Lagos Nigeria | 3 A&E Departments in London, Perth, Lagos  psychiatric attendances | Psychiatric emergencies (around Christmas and New year)  24.12.-03.01.  Comparison 1998/99 to 1999/2000 to 2000/2001  Total N not reported | NA | NA | -  (increase after holidays) | -  (increase after holidays)  Millennium increase in Lagos only |  | C |
| Ballard 1991^33^ | Coventry, UK  EU | Walsgrave psychiatric unit, catchment population 650’000 people. | Admissions (Christmas census)  Comparison week before Christmas to February  N=15 vs. 24.5 per week (98 per month) in February  1989 | NA | NA | Lower than in February | NA |  | C |

| Quality rating criteria: | - National or larger regional source of data vs. single or few centres |
| --- | --- |
|  | - Sufficient data collection duration (> 1-2 years) |
|  | - Clearly described outcome definition (suicide, self-harm, etc. how assessed) |
|  | - Completeness of reporting (total numbers, …) |

| Quality ratings: | - A (all criteria fulfilled) |
| --- | --- |
|  | - AB (one criterion not completely fulfilled) |
|  | - B (one relevant criterion not fulfilled) |
|  | - BC (more than one criterion not completely fulfilled) |
|  | - C (more than one criterion not fulfilled) |
